# Breaking New Ground With Endoxifen: Augmentation Strategies in OCD Management—A Case Series

**DOI:** 10.1155/crps/2908673

**Published:** 2025-03-26

**Authors:** Rishabh Singh, Markanday Sharma, Samiksha Sahu, Arka Adhvaryu

**Affiliations:** ^1^Department of Psychiatry, Command Hospital (Eastern Command), Kolkata, West Bengal, India; ^2^Department of Psychiatry, Military Hospital, Jhansi, Uttar Pradesh, India; ^3^Department of Psychiatry, Gandhi Medical College, Bhopal, Madhya Pradesh, India; ^4^Department of Psychiatry, RG Kar Medical College, Kolkata, West Bengal, India

**Keywords:** Endoxifen, impulsivity, obsessive–compulsive disorder (OCD), selective estrogen receptor modulators (SERMs), Yale–Brown obsessive–compulsive scale (Y-BOCS)

## Abstract

Obsessive–compulsive (OC) disorder (OCD) is a common and potentially disabling illness with a waxing and waning course. OCD significantly disrupts the quality of life. Selective serotonin reuptake inhibitors (SSRIs) are first-line pharmacological treatments for OCD and benefit up to half of the patients. Augmentation with low-dose antipsychotics is an evidence-based second-line strategy. Psychotherapy, including cognitive behavior therapy (CBT), is used both as first and second-line treatment. A significant portion of patients, however, do not respond to conventional treatments. We present a case series on the use of Endoxifen as an augmenting agent in patients with OCD and multiple psychiatric comorbidities who did not respond well to conventional pharmacotherapy.

## 1. Introduction

Obsessive–compulsive (OC) disorder (OCD) is characterized by the presence of obsessions, compulsions, or both. Obsessions are “repetitive and persistent thoughts, images, impulses, or urges that are intrusive and unwanted, and are commonly associated with anxiety.” Compulsions are “repetitive behaviors or mental acts that the individual feels driven to perform in response to an obsession according to rigid rules or to achieve a sense of completeness” [1]. It has a lifetime prevalence of 1%–3% [2]. Previous longitudinal studies reveal that only 20% of OCD patients achieve full remission [3].

A bidirectional relation is seen in OCD and other psychiatric comorbidities. On the one hand, OCD is associated with substantial psychiatric comorbidity, the commonest being anxiety disorders, mood disorders, impulse-control disorders, personality disorders, and substance use disorders (SUDs) [2, 4]. On the other hand, OCD has been frequently observed in patients with bipolar disorder. Epidemiological studies indicate that the prevalence of OCD among patients with bipolar disorder is notably higher compared to the general population [5, 6]. OCD frequently co-occurs with schizophrenia as well, with as many as 30% of individuals with schizophrenia reporting OC symptoms, and 12%–14% of these meet the diagnostic criteria for OCD [7]. Comorbid OCD and schizophrenia are associated with poorer outcomes, greater severity of psychotic symptoms, early age of onset, and longer days as inpatient treatment [8]. OCD prevalence in patients with SUD ranges from 6% to 12%, which is two to six times higher than those found in the general population. Conversely, lifetime SUD rates in individuals treated with OCD as a primary diagnosis range from 10% to 16%, which are slightly lower or comparable to SUD prevalence in the general population [9].

Managing OCD in the setting of other psychiatric comorbidities can be challenging. In comorbid bipolar disorder, the use of antidepressants (both selective serotonin reuptake inhibitors [SSRIs] and tricyclic antidepressants [TCAs]), which are the cornerstone of treating OCD, is avoided, as they may induce a switch to hypomania/mania [10, 11].

The worsening of OC symptoms with atypical antipsychotics in schizophrenia is well-documented, with clozapine being the most implicated agent, followed by risperidone and olanzapine. These second-generation antipsychotic-induced OC symptoms are predominantly observed in males in their 20s and 30s [12]. Additionally, a few case reports have suggested a potential link between aripiprazole and the de novo emergence of OC symptoms [13, 14].

Current clinical guidelines are agnostic about the use of SSRIs/TCAs in cases of OCD with comorbid bipolar disorder and SUD [11, 15]. Further complicating the situation is the recommendation of aripiprazole and risperidone as the agents of choice in cases where pharmacological augmentation is required, which are known to cause de novo/worsen OC symptoms in comorbid schizophrenia [11].

Endoxifen, a protein kinase C (PKC) inhibitor, is approved for use in manic episodes, with or without mixed features of bipolar I disorder in India [16]. The evidence for its effectiveness in disorders with some components of impulsivity, such as trichotillomania, borderline personality disorder (BPD), and SUD, is growing [17–19].

We present a case series describing the utility of Endoxifen in patients with OCD, along with other psychiatric comorbidities, who showed suboptimal response to conventional pharmacological agents recommended for OCD.

## 2. Case Illustrations

### 2.1. Case 1

A 38-year-old married male, with no family history of psychiatric illness, was diagnosed with OCD (International Classification of Diseases, Tenth Revision [ICD-10], F42) and was under treatment for OCD for the past 7 years. He was also diagnosed with alcohol dependence syndrome (ICD-10, F10.2) with continuous use. He experienced obsessive thoughts of contamination and engaged in compulsive cleaning and washing, which were associated with avoiding going outside and interacting with his family members, and spending a significant amount of time bathing. He was treated with Fluoxetine 40 mg/day, Olanzapine 20 mg/day, Fluvoxamine 200 mg/day for OCD, and with Naltrexone 50 mg/day as an anticraving medication for alcohol dependence. Despite being on multiple medications, he only had partial improvement in his symptoms, with some reduction in obsessive thoughts but persistent compulsive behaviors and distress. He had attended eight sessions of cognitive behavior therapy (CBT) previously, with minimal response.

Before starting Endoxifen, the Yale–Brown obsessive–compulsive scale (Y-BOCS) score was recorded as 34. He was administered Endoxifen 8 mg/day and reviewed monthly over the next 4 months. There was a subjective improvement in his symptoms, with the greatest improvement seen in the distress caused by obsessive thoughts and the time spent performing compulsive behaviors. The Y-BOCS score at the end of 4 months was 24.

### 2.2. Case 2

A 44-year-old married male, with no significant past or family history of medical, surgical, or psychiatric illnesses, was diagnosed with OCD (ICD-10, F42) and had been under treatment for the past 8 years. He had obsessive thoughts of blasphemy, with compulsive acts of asking for forgiveness and mental acts of offering prayer seven times. There was significant distress associated with obsessive thoughts along with avoidance of religious places and conversations. He was being treated with Fluvoxamine 200 mg/day, Escitalopram 20 mg/day, and Olanzapine 10 mg/day, with only partial relief of symptoms. He had also attended 12 sessions of CBT but with little success.

Before starting Endoxifen, the Y-BOCS score was recorded as 28. He was administered Endoxifen 8 mg/day and reviewed monthly over the next 6 months. There was a subjective improvement in his symptoms, with the greatest improvement in the distress associated with obsessive thoughts and degree of control over the obsessions and compulsions. The Y-BOCS score at the end of 4 months was 19.

### 2.3. Case 3

A 47-year-old unmarried male, with a family history of bipolar affective disorder in his mother, was diagnosed with bipolar affective disorder (ICD-10, F31) and OCD (ICD-10, F42). He had three episodes of mania and one episode of depression in the past; however, during the present evaluation, he had no mood symptoms. He was being treated with Divalproex sodium 1500 mg/day, Quetiapine 400 mg/day, Fluoxetine 20 mg/day, Haloperidol 10 mg/day, and Trihexyphenidyl 4 mg/day. He had OC symptoms for the past 15 years in the form of predominantly compulsive acts of cleaning the household and utensils, along with spending prolonged periods in the bathroom cleaning himself. Fearing contamination, he did not allow his family members to enter his room. He had poor insight into his illness and showed minimal resistance to his compulsions. He had attended 10 sessions of CBT for OCD but continued to experience severe compulsions with minimal symptom relief.

Before starting Endoxifen, the Y-BOCS score was recorded as 30. He was administered Endoxifen 8 mg/day and reviewed monthly over the next 6 months. There was a subjective improvement in his symptoms in the form of improved insight and a reduction in time spent performing compulsive acts. The Y-BOCS score at the end of 4 months was 23.

### 2.4. Case 4

A 33-year-old married female, with no family history of psychiatric illness, was diagnosed with schizophrenia (ICD-10, F20) at the age of 23 years. She also had comorbid OC symptoms for the past 3 years. She reported having recurrent, intrusive images of male genitalia coming to her mind whenever she would interact with a man, which would make her visibly distressed. To neutralize these thoughts, she performed compulsive acts such as shrugging her head and offering prayers in her mind. These symptoms impaired her social interactions, and she would often avoid attending social events or going outside her home. She was treated with Fluoxetine 40 mg/day, Imipramine 100 mg/day, Risperidone 4 mg/day, and Trihexyphenidyl 2 mg/day. She had previously attended seven sessions of CBT for OCD; however, despite pharmacotherapy and psychotherapy, she only had partial improvement in her symptoms.

Before starting Endoxifen, the Y-BOCS score was recorded as 36. She was administered Endoxifen 8 mg/day and reviewed monthly over the next 5 months. There was a subjective improvement in her symptoms in the form of a reduction in the frequency and distress associated with the images and consequent improvement in social interaction. The Y-BOCS score at the end of 4 months was 27.

## 3. Discussion

Endoxifen, which is a secondary metabolite of tamoxifen, is produced by CYP2D6-dependent biotransformation of the primary tamoxifen metabolite, N-desmethyltamoxifen. It exerts partial agonistic activity at estrogen receptors (ERs) and has been used earlier for managing ER + breast cancer [20]. Animal studies have previously revealed selective ER modulators (SERMs) such as tamoxifen display neuroprotective effects in the striatum of both male and female mice, possibly due to their antioxidant and free-radical-scavenging properties [21]. ERs (ER*α* and ER*β*) in the hippocampus modulate the release of brain-derived neurotrophic factor (BDNF) and neuropeptide Y (NPY), influencing neuroplasticity. These effects are observed not only in the hippocampus (affecting learning) but also in the amygdala (modulating aggression, anxiety, and fear) and the hypothalamus (regulating appetite, metabolism, and the hypothalamic-pituitary-adrenal axis). Additionally, ER activation promotes neurogenesis and exerts neuroprotective effects [22].

### 3.1. Neurobiological Basis of Estrogen Modulation in OCD

Brouillard et al. [23] demonstrated that females using oral contraceptives exhibited reduced cortical thickness in the ventromedial prefrontal cortex (vmPFC) compared to males. The vmPFC has been viewed as a center for fear regulation. Increased vmPFC thickness has been correlated with greater fear extinction, learning and recall, and less fear generalization, along with greater resilience following trauma exposure and remission in treatment-naïve OCD patients [23].

Studies indicate that estradiol modulates the serotonergic system through four mechanisms: (1) increasing serotonin synthesis, (2) reducing serotonin breakdown, (3) modulating serotonin receptors, and (4) increasing synapse assembly. These mechanisms collectively lead to increased serotonin release and sustained serotonergic neurotransmission, which may exert a protective effect against OCD [24]. All these factors underline the role of estrogen and SERM (Endoxifen) as a potential pharmacological agent in OCD.

### 3.2. Rationale for Using Endoxifen in OCD

#### 3.2.1. Existing Challenges in Treating OCD

OCD not responding to conventional pharmacotherapy has traditionally been managed through switching to an alternative pharmacological agent, increasing psychotropic doses for adequate duration, or augmenting treatment with antipsychotics [25]. Other pharmacological agents include glutamatergic agents (memantine, lamotrigine, topiramate, riluzole, and N-acetylcysteine) and serotonergic agents (ondansetron and granisetron). Other augmenting agents include mirtazapine and clomipramine [11]. Despite the availability of evidence-based treatment guidelines, OCD follows a chronic course with low remission rates, ranging from 16% in the first year to 42% after 15 years, underlining the tenacity of the illness [26].

#### 3.2.2. Compulsivity and Impulsivity in OCD: A Paradigm Shift

OCD has conventionally been viewed as a disorder driven primarily by anxiety avoidance and excessive self-control [27]. In recent years, however, a competing perspective has emerged that proposes two phenotypes driving the behavioral features of OCD: compulsivity and impulsivity. Traditional views posited that these two phenotypes were diametrically opposite and OCD was an archetypal compulsive disorder, whereas contemporary views argue the phenotypes to be overlapping in nature [28]. Several studies have demonstrated high impulsivity in patients with OCD [29–31].

#### 3.2.3. Endoxifen and Its Role in Modulating Impulsivity

Impulsivity is a prominent feature of bipolar disorder, as well as other conditions where Endoxifen has shown therapeutic potential, including BPD, trichotillomania, attention-deficit/hyperactivity disorder (ADHD), and SUD [32–35].

Endoxifen modulates impulsivity through its effects on the PFC. Impulse regulation is mediated by the PFC, and deficits in this region have been linked to dysregulated PKC intracellular signaling. Aberrant PKC activity is associated with impaired cognitive function and deficient impulse control. Endoxifen as a PKC inhibitor can be utilized for managing impulsivity. It exhibits a four-fold greater inhibitory effect on PKC than its parent compound, tamoxifen, achieves steady-state concentration within 2 weeks of administration, and has a favorable safety profile [36].

In the latest edition of ICD (ICD-11), OCD has been reclassified from “Neurotic, stress-related, and somatoform disorders” to “Obsessive–Compulsive and Related Disorders (OCRDs),” aligning it with the classification in the Diagnostic and Statistical Manual of Mental Disorders, Fifth Edition (DSM-5). Disorders such as trichotillomania and excoriation disorder, which were previously categorized under impulse control disorders in DSM-IV, have now been reclassified under OCRD in DSM-5 [37]. The rationale for classifying these disorders under OCRD includes (1) phenomenological similarities (major symptoms being repetitive thoughts and behaviors and a failure of behavior inhibition), (2) overlap in demographic features (age of onset, comorbidity, genetic influences), (3) common brain circuitry and neurotransmitter abnormalities, and (4) overlaps in treatment responses [38, 39]. These factors suggest a potential role for Endoxifen in OCD management.

## 4. Clinical Findings

To the best of our knowledge, this is the first case series to report the use of Endoxifen in OCD. In this regard, Elangovan et al. [40], in a case report published in 2023, described a 30-year-old female with OCD not responding to Fluoxetine (80 mg/day) and behavioral therapy, who achieved remission with Endoxifen (8 mg/day). In the current series, all the patients were having OCD who were on various classes of medications; however, they did not feel satisfactorily treated. One patient had comorbid bipolar disorder for which Endoxifen is an approved medication; another had alcohol dependence for which Endoxifen has shown positive outcomes. Patient-related data are summarized in Table 1. Side effects were few, including nausea and transient anxiety, but none severe enough to stop Endoxifen treatment.

**Figure 1:**
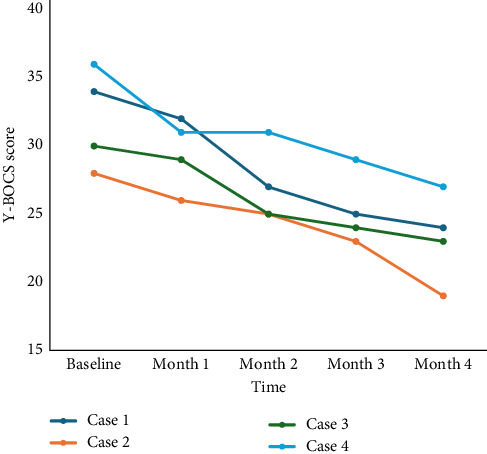
Y-BOCS score trend over time in OCD patients being treated with Endoxifen. OCD, obsessive-compulsive disorder; Y-BOCS, Yale–Brown obsessive–compulsive scale (the figure is created using Microsoft Excel).

The patients improved clinically as measured objectively by improvement in the Y-BOCS score over a period of 4 months. A paired *t*-test was conducted to compare the Y-BOCS scores before and after Endoxifen treatment. The mean difference was 8.75 (SD = 1.5), indicating a statistically significant reduction in symptoms (*t* [3] = 4.76, *p*=0.0008). Given the small sample size, a nonparametric Wilcoxon signed-rank test was also performed, which did not reach statistical significance (*p*=0.125). These findings suggest a potential benefit of Endoxifen in reducing OCD symptoms, warranting further investigation in larger cohorts. Case 2 showed clinical response with more than 35% reduction in Y-BOCS score [41]. We used Y-BOCS, a clinician administered scale, which is considered a gold standard in measuring the severity of OCD, has excellent inter-rater reliability, and high internal consistency [42, 43]. Trends of Y-BOCS scores over time are displayed in Figure 1.

## 5. Strengths and Clinical Implications

The study has several notable strengths. To the best of our knowledge, this is one of the first case series to explore the efficacy of Endoxifen in OCD patients unresponsive to conventional treatments. The study includes detailed case presentations, ensuring clinical applicability in real-world settings. Monthly follow-ups over 4–6 months provide longitudinal insight into treatment response trends. The Y-BOCS score trend analysis objectively quantifies symptom changes, adding to the robustness of the findings. The side effects reported were mild and transient, with no discontinuations due to adverse effects, reinforcing its potential safety profile in clinical practice.

The study explores biological mechanisms underlying Endoxifen's potential role in OCD, particularly PKC inhibition, which may reduce impulsivity and compulsivity, and ER modulation, which has emerging evidence in neuroplasticity and compulsive behaviors. This integrated molecular insight strengthens the scientific basis for using Endoxifen in OCD.

These strengths collectively position Endoxifen as a promising augmentation strategy for treatment-resistant OCD. Further randomized controlled trials are required to validate these preliminary observations and assess the long-term efficacy and safety of Endoxifen in this population.

## 6. Limitations and Future Directions

Despite its promising findings, this case series has several limitations. First, the study includes only four patients, which limits the generalizability of the findings. Additionally, the absence of a control group makes it difficult to determine whether the observed symptom reduction is attributable to Endoxifen or other confounding factors such as natural symptom fluctuation or pre-existing pharmacological treatments. The influence of psychotherapy (CBT) was also not systematically controlled, which may have influenced outcomes.

The study also carries a potential selection bias as the included patients were already receiving SSRIs and atypical antipsychotics, and the study does not include drug-naïve patients, limiting the generalizability of findings to the broader OCD population. Another key limitation is the heterogeneity in patient characteristics, as the sample includes individuals with comorbid psychiatric conditions such as bipolar disorder, schizophrenia, and alcohol dependence, making it difficult to isolate Endoxifen's specific effects on OCD pathology.

In terms of tolerability and safety, no major adverse effects were reported; however, side effect evaluation was based on subjective self-reports rather than systematic monitoring. Future studies should incorporate validated adverse event scales and objective monitoring tools, such as laboratory markers and ECG assessments, to assess the safety profile more comprehensively. Additionally, three out of four patients were on either risperidone or olanzapine, known to produce de novo OC symptoms. The drugs, however, were not stopped as patients preferred these medications, and their autonomies were respected.

From a statistical perspective, although the paired *t*-test demonstrated a significant reduction in Y-BOCS scores, the Wilcoxon signed-rank test did not reach statistical significance, likely due to the small sample size which may have increased the risk of Type II errors.

To address these limitations, future research should focus on randomized, double-blind, placebo-controlled trials with large sample sizes to establish causality and treatment efficacy. Studies with longer follow-up durations are needed to assess the long-term benefits and risks of Endoxifen in OCD. Moreover, stratified analyses based on comorbid psychiatric conditions could help determine whether certain patient subgroups respond more favorably to Endoxifen.

## 7. Conclusion

We presented a series of cases of OCD managed with Endoxifen as an augmenting agent. Treatment with Endoxifen was well-tolerated and resulted in improvements in Y-BOCS scores. Based on the observed cases, Endoxifen may reduce the severity of OC symptoms in patients who are resistant to conventional treatments. However, this hypothesis is exploratory and must be interpreted with caution, as it is derived from a limited number of cases without a control group, and further research is required to validate these preliminary findings.

## Figures and Tables

**Table 1:** Summary of patient characteristics included in the case series.

Patient parameters	Case 1	Case 2	Case 3	Case 4
Age	38	44	47	33

Gender	Male	Male	Male	Female

Comorbidities	Alcohol dependence syndrome	Nil	Bipolar affective disorder	Schizophrenia

Initial treatment	1. Fluoxetine 40 mg/day 2. Olanzapine 20 mg/day 3. Fluvoxamine 200 mg/day 4. Naltrexone 50 mg/day	1. Fluvoxamine 200 mg/day 2. Escitalopram 20 mg/day 3. Olanzapine 10 mg/day	1. Divalproex 1500 mg/day 2. Haloperidol 10 mg/day 3. Quetiapine 400 mg/day 4. Fluoxetine 20 mg/day 5. Trihexyphenidyl 04 mg/day	1. Fluoxetine 40 mg/day 2. Imipramine 100 mg/day 3. Risperidone 04 mg/day Trihexyphenidyl 02 mg/day

Duration of initial treatment (for OCD)	07 years	08 years	15 years	03 years

Previous psychological interventions (CBT)	08 sessions	12 sessions	10 sessions	7 sessions

Change in treatment	Endoxifen 08 mg/day	Endoxifen 08 mg/day	Endoxifen 08 mg/day	Endoxifen 08 mg/day

Duration of Endoxifen treatment	04 months	04 months	04 months	04 months

Number of follow-up sessions after initiating Endoxifen treatment	04 (once monthly)	06 (once monthly)	06 (once monthly)	05 (once monthly)

Y-BOCS score at baseline	34	28	30	36

Y-BOCS score after 04 months	24	19	23	27

Current status	Sustains improvement	Sustains improvement	Sustains improvement	Sustains improvement

## Data Availability

The data supporting the findings of this study are available from the corresponding author upon reasonable request. Due to privacy and ethical restrictions, the data are not publicly accessible.
